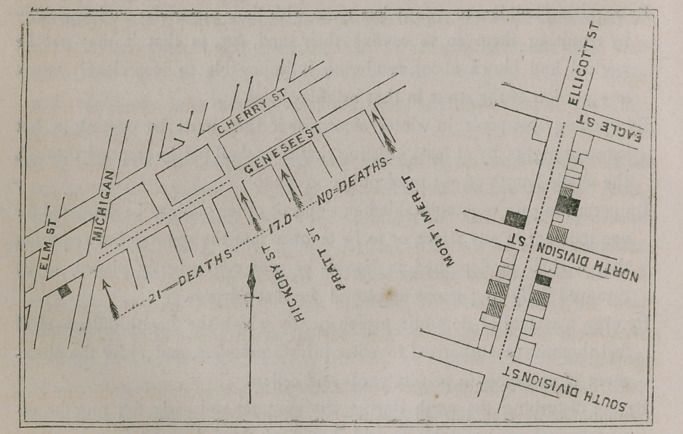# Report of a Committee Appointed by the “Buffalo City Medical Association,” August, 1852, to “Investigate the Influence of Upturning the Soil in the Causation of Cholera”

**Published:** 1852-10

**Authors:** 


ART. III.—*Report of a Committee appointed by the "Buffalo City Medical Association," August*, 1852, *to "investigate the influence of upturning the soil in the causation of Cholera." Read at a Special Meeting, held Sept.* 8, 1852, *and ordered to be published.*

GENTLEMEN OF THE ASSOCIATION:

The committee appointed by you at your last regular meeting "to investigate the influence of upturning of soils in the causation of the Asiatic cholera," would respectfully report.

*First.* That upturning of the soil is a sufficient and frequent cause of intermittents, and of other forms of malarious disease.

The correctness of this first proposition we presume no one will dispute; yet as it constitutes in some measure the basis of our argument, we think it proper to refer you to a few of the very common and notorious facts by which it is sustained.

The pioneer who builds his cabin in the wilderness seldom suffers in health until with his axe he has cleared away the forests from around his dwelling, and exposed the surface of the earth, covered with decaying vegetable remains, to the action of the air and of the sun.

In this instance the soil is not disturbed; but removing the forests which have for centuries buried the surface of the earth in its deep shadows, is the same in effect as bringing to the light a similar soil, long hidden, with the spade or the plough.

It is just as well known, however, that the emigrant who settles upon the prairie, lives in comparative health until the soil is broken; and that the surest protection against autumnal fevers is to place the garden and the ploughed fields remote from the dwelling. An extensive breaking of the fallow ground is almost invariably succeeded by a sickly season.

Among a multitude of local incidents which might be adduced to further corroborate our proposition, we will ask your attention to only that one which constitutes in part the subject of a memorial from the citizens of Rome, Oneida Co., to the Senate and Assembly of the State of New York: and especially to the letter accompanying the memorial, from Dr. H. H. Pope to Benjamin Enos, Canal Commissioner.

ROME, 30th December, 1843.

HON. BENJAMIN ENOS, Canal Commissioner, &c.

*Dear Sir* — By request of our citizens, I herewith transmit a list of such cases of sickness and deaths as occurred under my observation in 1842, the apparent result of the suspension of the then unfinished public works through our village: and I would first respectfully state, that the bed of the present enlarged canal, in most of its course, occupies the ground along side of the old Lock Navigation Co. Canal, and where most of the wood, timber, roots, and brush, graded from said canal was deposited. By excavating and exposing to the rays of the sun, this vast amount of decomposed and decomposing vegetable material, which had been covered in from five to eight feet of the swamp muck excavated from said canal some forty-five years since, a most prolific source of disease was produced. To the above, and the fact that in the partial excavation of the new canal previous to the suspension, the old canal was only partially filled up, leaving many places for pools of stagnant water, I attribute most of the sickness of that year: and by excavating and spreading over the vegetable matter above mentioned, the three or four feet of clean gravel and sand which forms the bottom of the bed of the enlarged canal, aided by the frosts of last winter, and the drain resulting from perfecting the bottoming out of the canal, I attribute our almost entire exemption from bilious disease the past summer. Should it be desirable, I might furnish a diagram of the lots along the canal, and the names of their occupants, and thus conclusively show that from ten to fifteen cases of disease occurred in the immediate vicinity of the canal, where one occurred in the upper part of the village.

I am, very respectfully, yours,

DR. H. H. POPE.

LIST OF CASES OF SICKNESS AND DEATHS.

Mrs. H. H. Pope and son, sister, and mother.
Mr. John Eddy's wife and two children.
Mr. Edward Eddy, wife, and family.
Mr. S. Hungerford and two children. One death.
Mr. Oliver C. Grosvenor. One death.
Mrs. R. Woldby, son, sister, and apprentice.
Mr. John Stevenson and family.
Mr. Luther Moltesar and family.
Mr. S. Martindale and family.
Mr. A. Seymour, wife and daughter.
Mr. W. T. Hungerford, wife, and son.
Mr. M. Rowley and family. One death.

Wm. Young—Joseph Shield's son, wife, and father. One death.
Mrs. Thomas Dugan and brother. One death.
Mr. Sandford Adams and wife. One death.
Mrs. Elonzo D. Lewis and family.
Mr. Steel, and wife and daughter. One death.
Mr. Snow—Mr. John E. Henderson and family. One death.
Mrs. McConich and child.
Mrs. Giles Hanby.

To this list I might add very many cases mostly of a mild form, which submitted readily to medical treatment; and I should also add, almost every Irish family whose shanty was situated in the immediate vicinity of the public work.—(Sen. Doc., No. 90, p. 35, April 15, 1845.)

The Hon. Henry A. Foster, senator, by whom the memorial was presented, and who was at that time a resident of Rome, informs your committee that all of this sickness occurred within twenty rods of the canal.

Confident, however, that you do not deny the competency of these causes in the production of fevers, we shall proceed to affirm

*Second.* In all these cases, where newly opened soil occasions fevers, it is the old and decaying vegetable matter thus brought to the surface, which chiefly or alone produces the diseases which result. (See Appendix, B.)

We believe that you will not demand of us an argument in defense of this position.

It is not quite certain, however, that aluminous soils freshly exposed, may not produce malaria; and if so we shall find, perhaps, an explanation in the fact that such soils generally contain more or less organic vegetable matter in an intimate state of mixture, especially where they lie underneath alluvium.

To be more definite, then, we affirm that the malaria, or disease-producing emanations from a soil freshly exposed, and the malaria from low and marshy soils are identical — the results alike of vegetable decomposition.

We shall have immediate occasion to apply this conclusion in considering the influence of upturning of the soil in the causation of Asiatic cholera.

*Third.* Marsh malaria produce not only intermittent fevers, and various zymotic diseases, such as yellow fever, typhus, &c., but also Asiatic cholera.

If this proposition shall be sustained, and our reasoning has been hitherto correct, then, by a plain rule of logic, is it at once established that upturning of the soil may produce cholera — the object of our commission is already reached and the question proposed by the "Association" determined.

Whether marsh malaria may develop cholera is a point in our argument upon which we might easily occupy much time, since the facts upon which its elucidation depends, cover the progress and history of this epidemic from the day of its first irruption from Jessore, in India, to the present time.

Here again, however, your committee finding the question already and now for a long time decided in the affirmative, do not feel at liberty to detain you by a lengthy parade of testimony. But as the latest and by far the most complete summary of the causes of the cholera is contained in the British Registrar-General's Report for 1848–9, we shall copy from a critical notice which we find in the July No. for 1852, of the British and Foreign Medico-Chirurgical Review, such facts and remarks as seem pertinent.

" Of all the causes influencing the spread and the mortality of cholera, none has so great an effect as elevation. This fact known for a long time," says the reviewer, " has been worked out by Mr. Farr so completely, that it may be received like the solution of a problem."

" The mortality from cholera is in the inverse ratio of the elevation. The mortality of the 19 highest districts was at the rate of 33 in 10.000; and of the 19 lowest 100 in 10.000." (Report.)

" On further examination," the reviewer concludes that in spite of other disturbing causes " the mortality from cholera bore a constant relation to the elevation." Density of population, over-crowding, poverty, general insalubrity, are all taken into the account as disturbing influences, yet by the side of elevation they have comparatively little effect, and not only in London but in the rural districts equally were the same conclusions obtained — still " in every place, elevation exerted a paramount effect."

Finally, Mr. Farr proceeds to explain how this happens:

" As we ascend, the pressure of the atmosphere diminishes; the temperature decreases, the fall of water increases, the vegetation varies, and successive families of plants and animals appear in different zones of elevation. The waters roll along the surface of the rocks, or filter through them and the porous strata of the earth, to burst out below—the sources of rivers or of tributaries, which carry disintegrated rocks with the remains and excretions of vegetables, animals, or men, in every stage of decomposition. The deposits in stagnant places, and at the estuaries, show the kind and quantity of mixed matter which the laden rivers carry down and deposit on the low margins of the sea at the tidal confluences of the fresh and salt waters. * *

" As the rivers descend, the fall of their beds often grows less, and the water creeps sluggishly along or oozes and meanders through the alluvial soil. The drainage of the towns is difficult on the low ground, and the impurities lie on the surface or filter into the earth. The wells and all the water are infected. Where the houses are built on hill-sides and elevations, as in London, the sewage of each successive terrace flows through the terrace below it, and the stream widens, the ground becomes more charged, every successive step of the descent, until it is completely saturated in the parts lying below the high-water mark.

" The river, the canals, the docks, and the soil of a port may be viewed as a large basin full of an almost infinite variety of organic matters, undergoing infusion and distillation at varying temperatures; and as the aqueous vapor which is given off ascends, it will be impregnated with a quantity of the products of the chemical action going on below, variable in amount, but necessarily greatest in the lowest and foulest parts. * * * The amount of organic matter, then, in the atmosphere we breathe, and in the water, will differ at different elevations, and the law which regulates its distribution will bear some resemblance to the law regulating the mortality from cholera at the various elevations. It has been seen how rapidly in London the mortality from chol era diminishes a few feet above the low ground on a level with the Thames, while several feet of elevation in higher regions produces no sensible effect. * * *

"It is established by observation, that cholera is most fatal in the low tow ns and in the low parts of London, where, from various causes, the greatest quantity of organic matter is in a state of chemical action; and it may be admitted that cholera, varying in intensity with the quantity, is the resultof some *change* in the *chemical action* of this matter. Further inquiry must determine whether in England that change is spontaneous, or the result of the introduction of a zymotic matter from beyond the seas; whether the poison enters the human frame in air or water, through the skin, the mucous membrane, or the air-cells of the lungs." (pp. 69–70.)—(p. 44.)

"This law of elevation," the reviewer remarks, "is perhaps the most important practical point brought out in the Report, and is well worthy the attention of the authorities of the East India Company; for the fact, though long recognized, has never been so definitely shown before." And he concludes by saying,

"The readers of our journal need scarcely be reminded how frequently we have advocated views identical with these, and how we have over and over again pointed out, that all observers who have regarded cholera with an unprejudiced eye, from the days of Jameson downward, have adopted opinions of a similar kind. Let us hope that this reiterated assertion—an assertion based on observations so numerous and so accurate, may at last have some weight with the rulers of this and other countries; and that we may at length commence in good earnest those works of sanitary improvement, the neglect of which is the opprobrium of the present generation, and the fatal legacy which it seems is to be inherited by the next." (p. 44.)

Your committee believe, and we think you will fully concur with us, that while the statistics and tables of Mr. Farr establish conclusively the *general* relation of elevation to the cholera, yet as the summaries only show the results of aggregates, it is very probable that among the materials from which the whole is gathered, might be found many exceptions. Our own experience would show that such exceptions do occasionally occur, in which eleva tion affords little or no immunity. The site of Bellary, in India, upon which an English fort is built, "is a granite rock, half a mile in diameter, and 500 feet in height. In its neighborhood are no marshes, no rivers, no dense and exuberant vegetation, which may afford to cholera a congenial soil; and yet, for thirty years, the pestilence has never been absent a single season from Bellary." (Trans. Amer. Med. Assoc. 1849. Report of committee on Med. Sci., p. 72.) But we shall find a sufficient explanation in other circumstances which are present. The barracks are over-crowded and uncleanly; close by are "two dirty bazaars, which have long been considered a public nuisance. *A large tank, which becomes dry during the hot season, taints the air with its effluvia.*" "When we take into account the climate of the country, it is not surprising that these causes are productive of cholera from one year to another. The heat during the months of March, April, May, and part of June, is described as being insufferable. The unclouded sun glares from a sky of brass upon the parched earth, and its fierce rays acquire additional force by being reflected from the granite rock; the thermometer rises, at midday, to 90°, and even 98° in the shade, and to 130° in the sun; and the heat is rendered more oppressive by the sultry stillness of the atmosphere. The winds which occasionally interrupt this calm, burn as if they had passed over a furnace, and are more intolerable than the still atmosphere itself." (Ibid p. 73.)

We would infer, therefore, that the condition of elevation upon which the immunity from cholera depends, is certainly not the diminished pressure of the atmosphere, to which Mr. Farr has, among other conditions alluded, since this condition is uniform. One other condition, however, we find here, which is unusual to such localities, and which is so constant in lower situations, viz., stagnant and putrid water. To this, therefore, in the present instance, most intense cause, with the concurrence of several other causes, such as idio-miasm, filth and excessive heat, your committee choose chiefly to refer the existence of the cholera at Bellary. It is the drainage, ventilation, and complete absence from malaria, which give to elevated situations their remarkable immunity; and where these accompanying conditions are absent, we venture to say, the same immunity will not be found.

For the same reason, also, the observation of Dr. Jackson, will be found generally true, viz., that the epidemic has never been destructive in granite regions; while others have remarked that in the spread of the cholera through the western states, "it has seemed to assume its highest malignancy in regions of country where the older limestone rock is the geological formation."

Granite regions are not generally miasmatic regions; but where lime-rock prevails the soil is mostly alluvial and is underlaid with clay, presenting thus the very conditions most essential to exuberant vegetation and to the detention of the water upon the surface. Lime-rock regions are therefore most often malarious, and, as others have observed, and as we have observed also, most often the chosen regions for cholera. Bellary is built upon a rock of granite, yet granite separated from those other conditions which elsewhere so almost universally accompany this formation, affords her no protection. Nor is it *necessary* that towns built in valleys, at the estuaries of rivers, or upon the margins of basins of water, or, indeed, wherever the geological formations are alluvium, clay, and lime-rock, should suffer perpetually the visitations of this pestilence; since money and labor, judiciously expended, may always conquer these natural disadvantages of situation.

But we need not look abroad for attestations of the fact that cholera seeks miasmatic regions. Its terrific assaults upon our neighboring towns, Sandusky and Toledo, in 1849, are well enough remembered. Sandusky, especially, was more than twice decimated. Nor have these places escaped during the present season. Wherever, also, in this state or in the neighboring states, in the city or the country, like causes exist and in equal intensity, there you shall learn that the cholera, true to its instincts, has not failed to pay its unwelcome visits; and if any places escape now that did not escape in the previous epidemics, it has been because some essential improvement has been made in their sanitary system, or in some way ancient malaria have been made to cease. Of this fact we might easily furnish you with several marked illustrations.

In our own city, each successive return of the epidemic has found it earliest and in greatest severity on the "flats;" in the vicinity of the canals, and stagnant pools which every where dot that portion of the town; at the "Hydraulics," and over that broad, level, yet quite *elevated* section which reaches eastward from Main street — throughout all of which localities there has always been, and there remains to the present day, when the cholera is not present as its substitute, intermittent fevers.

In other parts of the city, also, might be indicated many similar localities, which furnish similar evidence of their insalubrity; and investigations instituted by the vigilant officers who composed the "Board of Health" in 1849, seldom failed to discover, wherever the disease for a time fixed itself, an undrained cellar, an unfilled lot, an obstructed street gutter, or some like circumstance, as the source of the sickness.

It is not alone those, however, who live above or adjacent to these causes who are made to suffer; but if the winds are favorable, those living remote, and in the more salubrious portions of the town may fall under their poisonous influence.

So now there are operating about us, and surrounding us in such a manner that we cannot for our lives escape them, a thousand efficient causes, in all those standing pools covered with green fungi — of whose pestiferous agency few have thought, and for the removal of which none have cared.

We will not omit to mention, also, as having a clear relation to the point under consideration, that the cholera occurs oftenest in those houses which are destitute of cellars, and in which the floors are laid close upon the ground, or in dwellings the cellars of which are damp, not ventilated, or contain vegetables in a state of decomposition. (Appendix, C.)

Equally pertinent, also, is the well known fact that cholera, like malarial fevers, makes its attacks most frequently at night, or early in the morning, when the atmospheric vapors, holding miasmatic poisons in suspense are nearest the surface of the earth.

Those employed, therefore, in the excavation of alluvial earth during the day, have relatively little or nothing to fear — the vapors heated and rarified by the sun, arise rapidly and pass off. Hence the argument which has been occasionally urged before us, viz., that the workmen employed in these excavations do not fall sick, has no weight. The same is known to be true in relation to marsh fevers. It is the inhalation of the marsh atmosphere at night which proves so especially fatal.

There is more seeming value, however, in the statement that men have been known to work all night over these ditches and in the midst of beds of vegetable mould, who have yet continued in excellent health. Mr. Brick, the intelligent superintendent of the Buffalo Gas Works, assures us that he has thus employed a number of laborers during the present summer, where mould was three feet deep, yet they have escaped the cholera.

But may not these facts only corroborate what has been a generally received opinion, also, in relation to the influence of marsh miasms in the production of disease, viz., that the system is more susceptible to their influence during sleep, and that they are comparatively inoperative during wakefulness. The traveler on the Pontine marshes, in Italy, is cautioned constantly against yielding to the seductions of sleep, lest he should thereby fall a victim to the malaria, and medical men, with others, have long sanctioned the belief.

But if no such explanations could be offered of these apparent exceptions, their number is too small altogether to weigh a feather against the cumulative and overwhelming evidence opposed.

In short, we are now prepared to reaffirm that marsh malaria do prove an exciting cause of Asiatic cholera; and indeed, your committee are disposed to regard such malaria, if not the most energetic, at least as of all others the most widely operating cause.

We thus arrive again at the conclusion which we had already anticipated, and which constitutes our *fourth* proposition, viz: *That upturning of the soil may prove an exciting cause of Asiatic Cholera.* This conclusion, it must be seen, is the inevitable corollary from our argument.

We suppose that in the strict interpretation of the resolution under which we act, we might properly have here terminated our inquiries. But we believe that the spirit of your instructions extended farther — and that you proposed also, that we should inquire whether in *our city* there had been any cases in which the upturning of the soil had produced cholera; or whether, indeed, the soil was generally of that character from which in future danger might be apprehended, if the practice of opening and exposing the earth in the season of the cholera should be pursued.

To this subject we have therefore diligently addressed ourselves: yet we are forced to confess that here our labors have been less fruitful of results than we could have desired.

*First.* In relation to the cholera as it appeared during the last week in July, in Ellicott street, and its probable connection with the opening of a ditch in that street at about the same time, we find no farther room for investigation. The facts are before you in a report presented, at the regular meeting in August, by the chairman of this committee.

*Second.* In consequence of a reference made incidentally in the report just mentioned, to a similar occurrence in Genesee street, in 1849, we sought at once all the sources of information which at this late day were accessible, to determine the facts.

From Mr. Harraden, the contractor, we learn that a ditch was commenced in Genesee street, at the intersection of Michigan, about July 1, 1849, and that it was opened and completed through to Hickory by about the 20th of August; the work of opening, laying the sewer, and refilling, being carried on simultaneously — so that it was closed in its entire length very soon after the excavation was completed. The excess of earth was, however, not removed until about the 14th of September. The ditch was 1200 feet long, two feet wide and from eight to ten feet deep.

The soil through which the ditch was excavated was first one foot of paving sand, then clay to the depth of two or three feet, and finally hardened quicksand, or clay and sand in mixture.

From the files of the cholera reports kept in the office of the city clerk we ascertain that forty fatal cases of cholera were reported from Genesee street during the summer of '49; but in only three or four cases is the number of the house given, so that we were unable to determine from this source, the fatality of the disease along the line of the ditch. We will remark, however, that all the reports of fatal cases for this street were between the 19th of July and the 4th of September; a period wholly within that occupied in the opening and closing of the ditch.

We have also addressed circular letters to all those physicians, chiefly Germans, who we supposed had been the principal medical men employed in that district. But from none of them have we obtained any information which is of value, or pertinent to the question. Generally, they assure us they kept no records of these cases.

As the only remaining alternative, we secured the services of a very intelligent young gentleman, Mr. Augustus Jeyte, son of Dr. Jeyte, a German, who, at our request, promptly undertook to ascertain the facts by direct, personal inquiry. Accordingly Mr. Jeyte visited every house on Genesee street, between Michigan and Mortimer, and in the tabular form which we had arranged for him, reported to us the following results.

We give the tables as reported, that an opportunity may be afforded for correction, if there is any error. It will be seen that there are in all seventy-nine families referred to, of which twenty-four have removed, nearly one-third; among which number, doubtless, some sickness and deaths occurred. This must render the tables somewhat inaccurate, yet not sufficiently to affect materially their value.

In company with one of the aldermen of our city, the chairman of your committee has visited many of the dwellings, and we have confirmed, as far as we examined, the faithfulness of Mr. Jeyte's returns, with only two exceptions, which we have corrected. We also ascertained that from Michigan to Elm, the cholera occurred in but one family. In this family three died.

### FROM MICHIGAN TO HICKORY STREET—28 FAMILIES.

| No. | Name of Occupant. | Before July 1. | | Between July 1 and Sept. 14. | | After Sept. 14. | |
|---|---|---|---|---|---|---|---|
| | | Sick. | Died. | Sick. | Died. | Sick. | Died. |
| 157 | Changed occupant, | | | | | | |
| 158 | " " | -- | -- | -- | -- | -- | 2 |
| 159 | Lorenz Gillig, | | | | | | |
| 160 | Frederick Wuest, | | | | | | |
| 161 | Changed occupants, | | | | | | |
| 164 | " " | | | | | | |
| 168 | —— Loechler, | 11 | 2 | | | | |
| 169 | Changed occupants, | | | | | | |
| 171 | Peter Schmal, | -- | -- | 1 | | | |
| 172 | —— Reinald, | -- | -- | 3 | 1 | | |
| 174 | —— Wepner, | 1 | | | | | |
| 176 | —— Angelmiller, | -- | -- | 6 | 3 | | |
| 178 | —— Back, | -- | -- | 1 | | | |
| 179 | —— Spengler, | -- | -- | 9 | 4 | | |
| 180 | Changed occupants, | | | | | | |
| 181 | " " | | | | | | |
| 182 | " " | | | | | | |
| 184 | —— Webster, | -- | -- | 2 | | | |
| 188 | —— Washington, | 2 | | | | | |
| 189 | Wm. Messing, | -- | -- | 5 | 2 | | |
| 190 | —— Jackson, | -- | -- | -- | -- | 1 | 1 |
| 190 | Fried. Kibler, | | | | | | |
| 192 | Bodamers House, | -- | -- | 7 | 2 | | |
| 195 | Joseph Hartman, | 1 | | | | | |
| 199 | Andrew Guenther, | -- | -- | -- | -- | 2 | |
| 200 | Vincens Messmer, | | | | | | |
| 202 | John Sabel, | | | | | | |
| 206 | Joseph Ambs, | | | | | | |
| 209 | George Beckle, | -- | -- | 1 | | | |
| 210 | Mathias Lutz, | 1 | | | | | |
| 211 | John Wolf, | -- | -- | 1 | | | |
| 212 | Changed occupants, | | | | | | |
| 214 | Franz Droll, | | | | | | |
| 216 | —— Lutz, | | | | | | |
| 218 | Changed occupants, | -- | -- | 1 | 1 | | |
| 219 | Wm. D. Tute, | | | | | | |
| 225 | Friederich Emerich, | 4 | 2 | | | | |
| 228 | —— Bickel, | -- | -- | 1 | 1 | | |
| | | 20 | 4 | 38 | 14 | 3 | 3 |

FROM HICKORY TO EAST SIDE OF PRATT—10 FAMILIES.

| No. | Name of Occupant. | Before July 1. | | Between July 1 and Sept. 14. | | After Sept. 14. | |
|---|---|---|---|---|---|---|---|
| | | Sick. | Died. | Sick. | Died. | Sick. | Died. |
| 233 | G. Fisher, | -- | -- | 5 | 4 | | |
| 234 | Chrishtopher Schmahl, | -- | -- | 3 | 2 | | |
| 237 | —— Bauer, | -- | -- | 8 | 6 | | |
| 238 | Changed occupants, | | | | | | |
| 239 | —— Schandall, | -- | -- | -- | -- | 2 | 1 |
| 241 | Changed occupants, | | | | | | |
| 242 | " " | | | | | | |
| 243 | —— Uebelacher, | | | | | | |
| 244 | John Dechent, | | | | | | |
| 246 | —— Hauenstein, | -- | -- | 1 | | | |
| 248 | Nicholas Brick, | -- | -- | 5 | 2 | | |
| 248 | —— Klotz, | -- | -- | 4 | 1 | | |
| 250 | Frederick Geib, | -- | -- | 4 | 1 | | |
| | | | | 30 | 16 | 2 | 1 |

FROM EAST SIDE OF PRATT TO MORTIMER—17 FAMILIES.

| No. | Name of Occupant. | Before July 1, Sick. | Before July 1, Died. | Between July 1 and Sept. 14, Sick. | Between July 1 and Sept. 14, Died. | After Sept. 14, Sick. | After Sept. 14, Died. |
|---|---|---|---|---|---|---|---|
| 254 | Peter Seibert, | | | | | | |
| 255 | Changed occupants, | | | | | | |
| " | " " | | | | | | |
| " | " " | | | | | | |
| " | " " | | | | | | |
| " | " " | | | | | | |
| " | " " | | | | | | |
| 256 | " " | | | | | | |
| 257 | Adam Schauf, | | | | | | |
| 259 | Philip Schauf, | | | | | | |
| " | Jacob Scheu, | | | | | | |
| " | Joseph Meyer, | | | | | | |
| " | Valentine Schneider, | | | | | | |
| 262 | Changed occupants, | | | | | | |
| " | " " | | | | | | |
| " | " " | | | | | | |
| 277 | Adam Guth, | | | | | | |
| 279 | Wm. Borger, | | | | | | |
| " | Heinrich Thal, | | | | | | |
| " | H. Ekel, | | | | | | |
| 284 | Andres Bodamer, | -- | -- | 1 | | | |
| " | Changed occupants, | | | | | | |
| " | Christian Dismar, | | | | | | |
| 286 | Wm. Bedinger, | | | | | | |
| 287 | Anton Trot, | | | | | | |
| 288 | Christian Vogel, | | | | | | |
| 289 | F. Jacob, | | | | | | |
| 295 | Reinhardt Philip, | | | | | | |
| | | | | 1 | | | |
| | Total from Mich. to Mort., | 20 | 4 | 69 | 30 | 5 | 4 |

The summary may be stated as follows:

Whole number of cases from Elm to Mortimer during the season, 97. Whole number of deaths, 41. Of the deaths, 3 occurred between Elm and Michigan, 21 between Michigan and Hickory, 17 between Hickory and east side of Pratt, and none between east side of Pratt and Mortimer. Again, of the deaths, 30 occurred between the 1st of July and the 14th of September; four before the 1st of July, four after the 14th of September, and three are not determined. (See diagram in Appendix, A.)

It will not escape your observation that nearly all the deaths were along the line of the ditch, or within 300 feet of its north-eastern extremity, in which direction our winds would be most likely to carry the miasms. If the water courses were obstructed also, the refluence would be in the same direction, as the street has a declination from east to west and south.

Such are the facts as near as we have been able to ascertain them; and while your committee do not regard such evidence as conclusive, yet, when taken in connection with all the circumstances, we cannot avoid a belief that to the presence of the ditch ought to be ascribed in some measure the extreme malignancy of the cholera in its neighborhood.

Whether its agency depended upon direct emanations from the upturned soil, or upon the obstructions caused by it to the water courses, we cannot positively determine; yet, considering the character of the earth disturbed, we think the latter altogether the most probable supposition. The malaria from a soil like that through which this ditch was carried must have been, we apprehend, too inconsiderable to be regarded as in any manner an adequate source of the sickness.

Attempting to carry our investigations into other streets, through which ditches were opened during the same season, and in which it had been said that similar consequences had followed, we found our inquiries ending in no satisfactory results, and we therefore soon ceased our examinations. Ditches were made generally for the purpose of removing nuisances, in many streets, and in one instance at least, by request of the inhabitants. Such was the fact in Cherry street. The street was covered in various places with stagnant pools of water, and the lots had no means of drainage. In this condition the cholera broke out among the inhabitants, and they soon petitioned the Common Council to have a drain built in the hope that the disease might be thus arrested — but the cholera continuing to increase in severity after the work was commenced, and the completion being somewhat delayed, they again petitioned hastily to have it closed.

In this instance, also, if the opening of the earth had any connection with the increase of the cholera, it was probably in consequence of its increasing the water accumulations along the street.

If we had been disposed, we might have prosecuted our inquiries upon a larger scale, by an examination of the influence of the removal and deposit of many acres of earth upon the "flats;" a circumstance which has resulted from the necessity of lifting that portion of the town to protect it from inundations, and from the excavations of the Hamburg street canal, and the Ohio basin. But we are persuaded that no conclusions of value could be drawn where so many modifying circumstances need to be taken into the account. Here was originally a marl covered with vegetable mould, and it is well known to have been a source of malaria. It is now in part the same, and in part it is composed of irregular basins of clay, containing pools of water, and we presume, also, that it is now, as before, a source of malaria: but to what relative or actual extent we have not the means of determining. One thing at least must have greatly favored this district during the present season, viz., the general prevalence of south-west winds, which, while they have swept off the miasms generated upon the flats, may possibly have contributed materially to the greater fatality of the cholera in the north-eastern portions of the town, over which these winds must pass, bearing their vapors and their poisons.

To us it seems that while few cities have grown as rapidly as Buffalo, in few cities, also, has it become necessary to the same extent to disturb the soil for the purposes of grading, filling, paving, ditching, &c., &c., a circumstance which might have proved harmless where sand was the earth removed, but which, as we have shown, must be directly or indirectly a source of disease where clay and alluvium preponderate.

*Recapitulation.*—The conclusions to which we have arrived then are,

*First.* That upturning of the soil, under certain circumstances, will produce certain forms of disease, such as intermittents, &c.

*Second.* That in all these cases where newly opened soil occasions fevers, it is the old and decaying vegetable matter thus brought to the surface which chiefly, if not alone, produces the diseases which result.

*Third.* Decaying vegetable matter may produce not only fevers, but also, probably as an *exciting* cause, Asiatic cholera.

*Fourth. That as an inevitable inference, upturning of the soil may, in the same manner, produce Asiatic cholera.*

*Fifth.* That in our own city there are many localities under which vegetable soil has been buried; and that in all those parts the exposure of this under soil to the air may become an exciting cause of cholera.

*Sixth.* That upturning of a clay, or sandy soil, which is impregnated with vegetable matter, may also, in proportion to the amount of such organic materials contained, prove a source of cholera.

We wish it to be understood, however, that we regard this cause as quite, inconsiderable in the great majority of cases.

*Seventh.* That independent of the nature of the materials composing the soil the obstructions occasioned thereby to the free passage of water along the gutters, renders upturning of the soil indirectly a source of cholera; and not alone in the manner now indicated, but also by forming irregular basins which contain water, as in many ploughed and unpaved streets, and upon the "flats."

*Finally*, the practical inferences to be made from all we have said, are,

An active, and intelligent Board of Health, with the co-operation of the Mayor and Common Council and citizens, can do more for the arrest of the Asiatic cholera, than the most able body of medical men: since with them rests the task, so comparatively easy, of removing the causes; while with physicians only remains the work, so often impossible, of applying the remedy.

To this end, and that our city may become again as salubrious as it was known to be before its rapid growth had turned its pastures into pools, and its streets into muddy sewers—as healthy, and as free from epidemics as Utica, Schenectady, Troy, Albany, or indeed as any of our older sister cities, which are now enjoying an enviable exemption from the present epidemic; and for which they are indebted, no doubt mainly, to their complete drainage, and to the perfection of their public works, such as grading, paving, sewerage, &c.—to this end, we repeat, it is necessary that no narrow system of party interest, or mean economy should prevail; but influenced by enlarged views, which will esteem the lives of our fellow-citizens paramount to all other considerations, pecuniary or political, a plan must be devised, and a work carried out commensurate with the magnitude of the evil to be abated.

Were it necessary, however, we might easily demonstrate that even in a pecuniary point of view, the owners of property would find such expenditures remunerative. The reputation of increased salubrity which our town must soon enjoy, would, we have no doubt, return a quick interest for every dollar thus laid out.

By such measures as the following are these results only to be attained:

By a system of sewerage, perfect and coextensive with the city limits, or at least with occupied dwellings. (Appendix, D.)

By draining every cellar, and every lot whose level is below the street, or

By filling up vacant and undrained grounds.

By paving every street and lane, at least in those portions of the town where clay or alluvium preponderate.

By removing all obstructions which arrest or retard the flow of water through the gutters. For it ought to be strongly impressed, that a very small basin of stagnant water is, at certain seasons, the source of a very large amount of poisonous malaria.

By sweeping the streets *thoroughly*, once or twice a week; and not allowing the dirt to be again sifted and scattered along the way by the loose wagons with which its removal is attempted.

By requiring builders to occupy less room with their materials; and especially by requiring them so to confine their sand, &c., as that it may not be worked and blown about, rendering it impossible to keep cleanly swept any portion of the street in that neighborhood.

By opening cess-pools in winter alone; or if they must be opened in hot summer nights, by at least insisting that they shall not be emptied through the whole length of our most public avenues.

By permitting no vegetables, kitchen slops, or other offal, to be deposited for one moment in any street, or to be thrown into the sewers; but requiring that it shall be deposited in barrels or tubs conveniently placed, to be removed daily by persons employed for that purpose.

By even a system of domestic espionage, by which the health officers shall feel themselves authorized to enter private premises, and order the abatement of nuisances in private yards and cellars.

By not disturbing the earth during the summer and early fall months, especially where vegetable mould has been buried, unless upon urgent necessity; and where the necessity actually exists,

By returning the earth to its place again before nightfall, and at no time permitting it to obstruct the gutters, or water courses.

And if hereafter the cholera shall return to us, it will, we trust, find only here and there a victim, chosen from among that class whom poverty, or "over-crowding," or habits of persona filth, or intemperance, or other depressing vices, or habitual and gross imprudence, have most eminently predisposed to the disease. The causes will be confined to every man's own dwelling, and to every man's own self; and against such causes, thank God, we who are above want, may provide.

All of which is respectfully submitted.

FRANK H. HAMILTON, Chairman,
PHINEAS H. STRONG,
CHARLES H. WILCOX,
*Committee.*

### APPENDIX,

BY FRANK H. HAMILTON, M. D.

### A.

MAP OF PORTIONS OF GENESEE STREET AND ELLICOTT.

-------- Ditches.

Houses in which deaths from cholera occurred, are shaded black.

Houses in which cases of cholera occurred, but no deaths, are shaded in half tint.

### B.

The agency of decaying *animal* matter in the production of malarial fevers is pretty universally denied; and we regard, also, the evidence of its agency in the production of Asiatic cholera as quite equivocal. The odor from putrid animal substances, is certainly far from being agreeable, and we would choose always not to be obliged to respire it; yet every one knows that the offensiveness of an odor is no test of its unwholesomeness. Few odors are more offensive than sulphuretted hydrogen, yet at Avon at least, it is regarded as innocuous — on the contrary, it is snuffed up by the valetudinarians from early morning until night, as the very essence of life and health.

Decaying animal matter may, we know, under circumstances of extraordinary concentration, produce asphyxia, and possibly, yet of this we are not quite certain, low typhoid fevers: but no evidence of its competency to the production of malarial diseases or of cholera has, to our knowledge, ever been furnished. It is, therefore, that in our report we declare decaying *vegetable* matter to be, in our opinion, where earth is upturned, the chief or sole cause of the cholera which may result.

In confirmation of this opinion, we refer our readers to the very able and elaborate work on "Hygiene Publique," by Parent Duchatelet, published in Paris in 1836. The work is in two vols. octavo, and comprises over 1200 pages.

M. Duchatelet, formerly a physician, had devoted fifteen years of his life exclusively to the prosecution of the subject of Public Hygiene. He was also for many years one of the most active members of the "Conseil de Salubrité," and his reports, in reply to the various inquiries of the government, are very numerous, and are esteemed every where as authority. It will be understood that the "Council of Health," in Paris, is usually composed of the most distinguished members of the medical profession, who hold their places for a succession of years; and such confidence have the municipal government in their counsels, that seldom or never has their advice been rejected.

M. Duchatelet himself visited repeatedly all the workshops of the tanners, the manufacturers of animal grease, glue, music strings, Prussian blue, the slaughter-houses, and especially Montfaucon, that immense establishment for the disposal of old and worn-out horses. Here annually are brought, either dead or alive, from 12 to 13,000 horses. Every portion of this carrion is worked over and used up for various economical purposes. The main and tail, the hides, the meat, the bones, the hoofs, the shoes, the fat, the intestines, and their contents even, are piled into separate heaps, and worked up, or immediately sold. Nothing can exceed the filthiness of this "Chantiere dé Equarrisage." The enclosures and the air of the country for some extent around, are saturated with the most disgusting odors. Yet M. Duchatelet has ascertained that the laborers enjoy both here, in the tanneries, and in the manufactories of gut strings, as good health as others of the same class in other occupations. He tells us of the "fecondité remarquable" of the women, and of the "strength and "good looks" of the children, who are often cradled as it were "in the interior of a carcase." But what we shall notice in this connection as most curious and pertinent, is that all families in these various establishments enjoyed, says M. Duchatelet, *a remarkable exemption from illness during the destructive prevalence of the Asiatic cholera in Paris.*

We have ourself, in 1844, visited the same establishment, now removed to a greater distance from the city, for the purpose of verifying the observations of M. Duchatelet, and we can attest that among the children employed in picking over and assorting the various offals, we saw no indications of bad health, except the pallor which would naturally result from confinement and sedentary habits; for these children, male and female, sit in the midst of large heaps of offal, upon benches or stools, and thus are employed from morning until night. We can certainly attest that nothing could exceed the offensiveness of the odors with which the building, and the grounds, shut in by a high wall, were impregnated.

"M. Duchatelet," says the reviewer, "examines at great length the question, whether the existence of anatomical amphitheatres in a city is at all injurious to the public heath; as some years ago, it was often urged against these establishments, that they were so many 'foci' of infectious emanation. He has quite satisfied himself that there is not a shadow of truth in this statement; and among other arguments, he appeals with confidence to the healthy state of the Hotel Dieu in former years, when this hospital was surrounded by numerous dissecting rooms, some of which were even within its walls. He next adduces the testimony of a great number of eminent authorities to confirm his opinion. The first he quotes is the late M. Lallemand, who has left us an account of the dissecting rooms first established by Dessault. They were, it appears, situated on the top story of an old decayed house. The number of bodies usually on the tables was from fifty to sixty, and the number of pupils 200 or more. The rooms were very seldom cleaned, and even the debris of the bodies was not removed oftener than once a month. Nothing could exceed the abominable stench diffused over the immediate neighborhood; and yet, says M. Lallemand, we never heard of any diseases, which might be fairly attributed to the presence of the dissecting rooms, either among the students themselves, or among the inhabitants of the adjoining houses. Dessault himself used to say, that he really believed that the *odorous* air of his dissecting rooms saved him from attacks of epidemic and other disorders, of which those hospital physicians and surgeons, who seldom or never dissected a dead body, seemed to be much more susceptible than himself. He took pleasure in repeating the old saying—'morte la bete, mort le venin;' and therefore he did not feel at all desirous, he said, to alter the state of the rooms under his charge.

"M. Duchatelet next appeals to the testimony of Dubois, Dupuytren, Boyer and of a host of others still alive, who have been, or still are teachers of anatomy. They all agree in opinion, that it is quite an error to suppose that the air of a neighborhood is ever contaminated—so as to induce disease—by the emanations from dissecting rooms, or that the students ever suffer from breathing the impure air of these places.

"M. Duchatelet applied to M. Andral, among many others, for his opinion on this subject. The following is an extract from his letter:—'As to the diseases which medical students contract during their dissections, we have no good grounds for attributing them to cadaveric emanations. Gastroenterites, meningites, and typhoid fevers, are very frequent among the students during the first year of their stay in Paris; but these diseases depend so little upon the mere circumstance of the 'sejour' of the students in the dissecting rooms, that they are more common among those who have not begun to dissect. Indeed the mortality among the medical pupils is certainly not greater, than among an equal number of any other class of young men. The fatigues of study, the late hours, the intellectual exertion at home for the concours, &c., are much more hurtful to the constitution, than the labors of the dissecting room. I have taken the trouble to ascertain the general health of the servants of the amphitheatres—and some of them pass day after day there without once going out—and it appears that they are quite as healthy in every respect, as other men.'

"With respect to the health of the students themselves, M. Duchatelet has taken great pains to discover, whether there are any good grounds for supposing, that it is affected by the air and the labor of the dissecting room. According to his account, the most frequent complaint among these youths is dyspepsia, attended often with colicky pains and diarrhœa. But these symptoms are, says he, attributable much less to their engagements in the dissecting rooms, than to the imperfect or improper diet, which the 'res angusta domi' imposes upon many of them. Not a few of the French students, especially those who arrive from the provinces, are but scantily provided with even the absolute necessaries of life. M. Duchatelet assures us that he has known several, who have lived for weeks, aye, and months too, upon stale bread, and an occasional glass of brandy.

"It may be worth while to notice, also, the usual good health of the servants of the anatomical theatres. Many of them live within the premises; yet they do not suffer any direct inconvenience; and it has been often remarked that these men are singularly exempt from febrile diseases." (L. M. C. R. vol. xxvi. pp. 424–26.)

M. Rousseau, who for thirty-six years had superintended the dissections in the extensive rooms devoted to comparative anatomy, connected with the "Garden of Plants," declares to M. Duchatelet that neither he nor any of his assistants had ever suffered from their occupations even during the summer, although the bodies of the animals were often in a highly putrid condition.

Mr. Lawrence, of London, also confirms the same opinion, in a letter to Dr. Bancroft, quoted by M. Duchatelet, and cited by Dr. Warren, in the Boston Med. and Surg. Journal.

With such testimony before us, and with no knowledge of any conflicting evidence, we are forced to the conclusion that decomposing animal substances are not often a source of disease, and especially of the epidemic forms.

If, however, we find that vegetable decomposition is in some way the general cause of malarial diseases, and not unfrequently of cholera, dysentery, &c., yet we do not affirm our belief in any particular doctrine of malaria. In what the morbific agent consists to which, after the Italians, writers generally have applied this term, we have as yet no positive knowledge. It may be of a "fungous" or "cryptogamous" nature, a poisonous vegetable which innoculates itself upon the animal; it may be "gaseous," the result of a chemical separation and decomposition, or "animalcular," or any thing else upon which it is easy to speculate, but impossible to determine. The general concurrence of certain diseases, including epidemic cholera and dysentery, with the rapid decay of vegetable substances is all we pretend to assert: a concurrence which is sufficiently constant to furnish strong presumptive evidence that they stand in the relation of cause and effect. Nor is our confidence in this belief at all shaken by the facts stated by Linnæus in his thesis entitled "Hypothesis nova de febrium intermittentum causa," or by the observations of Von Aenvank, or of Bell, all of which establish that periodic fevers are common where an argillaceous or clayey soil prevails, as well also as in alluvial districts. On the contrary, such facts and opinions only confirm the doctrines to which we hold, and which we now advocate.

### C.

The following are briefly the circumstances, so far as we can ascertain them attending the sickness in the house No. 513, east side of Main street, to which the attention of the "Association" has been called by a verbal statement. The house is a very old frame building, composed of four or five rooms, situated in a healthy neighborhood, and with neat, clean premises.

The occupants were John Nugent, wife and infant — Irish. Mr. Wilson, wife and two children — English, and Sarah, an adult Irish girl.

John Nugent and Wilson were carpenters, and worked under cover: both temperate, steady, and healthy — and the same was the fact with all the family.

John Nugent was attacked Thursday, September 2, 1852, at 6, A. M., and died at 11, P. M.

Wilson was attacked at 3½, P. M., September 3, (Friday,) and died at 2, A. M., of Saturday.

Catharine, wife of John Nugent, was attacked Saturday (4th,) and died at the hospital Sunday, P. M.

Sarah was attacked after reaching the hospital Sunday (5th,) at 10, A. M., and died there the same day.

John Nugent's infant was attacked Sunday and died several days after.

Wilson and family had occupied the house three weeks; Nugent and family one week, and Sarah had been in the house, when she died, three days.

Having made careful inquiry, we learned that there had been no imprudence in diet.

Impressed with the belief that some local cause existed in or about the house to which the sickness was due, we inquired whether there was a cellar, and learned that there was a hole called a cellar, under the front room, but that Mr. Wilson having found it "close and musty" when they first occupied the house, they had determined not to use it, and had left it closed from that time.

Dr. Merriam, who was in attendance also, and the writer, immediately descended into the cellar, which was entered by a trap door from the dining room.

The air was very warm, damp, and suffocating. The soil in which the cellar had been made was clay; and the sides had been originally lined and walled up completely with boards. Most of these boards, however, had decayed, and the earth had fallen in, leaving one or two openings where some air and light were admitted. Nearly all the boards, in addition to being very much rotted, were covered with thick masses of vegetable mould, or fungi, of the cryptogamous order.

It is, perhaps, rather a curious than practical fact, that a careful microscopic examination made by Dr. Merriam, showed ten different species of cryptogamæ.

We did not doubt that in the vegetable malaria of this cellar we had found the source of the disease.

### D.

In many respects our system of sewerage is eminently defective. The mains are not sufficiently large — the branches are not sufficiently numerous, but especially is it defective in the almost complete absence of stench traps, which alone, we are informed, can effectually prevent the return of the gases into the streets and dwellings.

This is a great and rapidly increasing evil, and while we wish to give due prominence to the other causes, and especially to the surface and underground miasms, of which we have now spoken at length, we deem it proper to refer also to these sewer miasms as, in our city at least, an equal, possibly it may be, a much more abundant source of disease. It is the more proper to speak of them in this report, because it not unfrequently happens that in upturning the earth to lay gas and water pipes, the inlets or mains of the sewers are cut across and their gases thus escaping may produce those diseases which result. Such, however, we ought to say, was not the fact in Ellicott street, nor in Genesee and Cherry street. In Ellicott the inlets were below the bottom of the ditch, and in Genesee street and Cherry, the excavations were for the purpose of building sewers.

These sewers receive a large amount of offal, chiefly vegetable; and to be convinced that they may eructate villanous odors, one has only to place himself over one of the open mouths, or inlets, at any corner of the streets.

Most of the main trunks have their outlets above the surface of the water, on the creek or canals, open toward the prevailing winds, so that they are daily and hourly, when the winds are favorable, blowing their impurities up into the houses and into the streets. *At all times,* whether the wind favors or not, they do not cease to breathe out poisons and dispense it silently and imperceptibly into our dwellings. If we do not always recognize it, it is because we have in general become too much accustomed to it.

We are informed by Mr. Brick, that his men often fall sick and are obliged to leave their work when accidentally or by necessity, they tap one of these inlets or house drains.

That we should continue to be exposed to this source of disease, and of cholera no doubt, where the remedy is so easy, implies great neglect, if not actual criminality, on the part of our city authorities, as well as of the citizens themselves. Stench traps, constructed at a trifling cost, have been proven to be an effectual remedy. At the expense of the city they should be immediately placed at every street inlet; and no person ought to be permitted, under a suitable penalty for the omission, to connect his premises with a sewer without a similar sink. The expense to the city of keeping the street traps sealed by a sufficient supply of water from the hydrants, would be inconsiderable.

How effectual these stench traps prove, a large experience in other cities has fully determined, and some of our citizens have ascertained by having laid them in their own premises. But many of you, perhaps, will remember that when the gas works were first completed in the fall of '48, our whole town was made to celebrate the event by a sudden irruption into every house and street having a drain, of an intolerable odor of sulphuretted hydrogen.

The refuse gas from the gas works' establishment had been emptied by a chimney into one of the main sewers near its outlet, and a favorable wind had carried it upward into every artery of the city sewers, and people were driven from their houses into the streets, and even there the stench followed them.

Immediately, by order of the superintendent, Mr. Brick, the main sewer was cut off above the point where the gas pipe connected with it, and a stench trap was laid, and the offence at once ceased.

Something might also be accomplished, and probably at a cost to the city of less than $300 annually, by flooding the sewers occasionally during the summer season by turning several hydrants simultaneously into a single main.

## Figures and Tables

**Figure f1:**